# Decoding Hepatocellular Carcinoma Metastasis: Molecular Mechanisms, Targeted Therapies, and Potential Biomarkers

**DOI:** 10.3390/cimb47040263

**Published:** 2025-04-08

**Authors:** Ke Wei, Chunxiu Peng, Yangzhi Ou, Pengchen Wang, Chenjie Zhan, Huaxiu Wei, Jintong Na, Zhiyong Zhang

**Affiliations:** 1 State Key Laboratory of Targeting Oncology, National Center for International Research of Bio-Targeting Theranostics, Guangxi Key Laboratory of Bio-Targeting Theranostics, Collaborative Innovation Center for Targeting Tumor Diagnosis and Therapy, Guangxi Talent Highland of Major New Drugs Innovation and Development, Guangxi Medical University, Nanning 530021, China; 202221579@sr.gxmu.edu.cn (K.W.); 202410267@sr.gxmu.edu.cn (C.P.); 202321617@sr.gxmu.edu.cn (Y.O.); 15056984982@163.com (P.W.); 202221564@sr.gxmu.edu.cn (C.Z.); 202321625@sr.gxmu.edu.cn (H.W.); 2 Department of Surgery, Robert-Wood-Johnson Medical School University Hospital, Rutgers University, New Brunswick, NJ 08901, USA

**Keywords:** hepatocellular carcinoma, metastasis, molecular mechanism, signaling pathways, targeted therapy, biomarkers

## Abstract

Hepatocellular carcinoma (HCC) is a leading cause of cancer-related mortality worldwide, with metastasis representing a pivotal factor in poor prognosis and high fatality rates. This review offers a comprehensive examination of the key molecular events and regulatory mechanisms driving HCC metastasis, with a particular focus on genetic mutations, epigenetic alterations, and dysregulated signaling pathways. Special emphasis is placed on the role of three-dimensional genome structural remodeling in HCC initiation and metastatic progression. Additionally, the latest advances in targeted therapies for advanced HCC are summarized, including both first-line and second-line treatments, highlighting their impact on controlling metastatic disease. The review also examines a variety of potential biomarkers linked to HCC metastasis, including circulating tumor cells, circulating tumor DNA, and exosomal contents, all of which demonstrate significant promise for the early detection, diagnosis, and therapeutic monitoring of HCC metastasis. By bridging molecular insights with clinical applications, this review provides valuable perspectives to guide future research in the diagnosis and treatment of HCC metastasis.

## 1. Introduction

According to the “Global Cancer Statistics 2022”, liver cancer ranks as the sixth most prevalent cancer globally, while its mortality rate stands at third place, with hepatocellular carcinoma (HCC) accounting for the majority of cases [1]. Up to 60% of patients with locally resectable disease survive for five years post-diagnosis [2]; however, the survival rate for those who develop distant metastasis drops to a mere 4% [2]. The metastasis of HCC remains a critical factor in significantly reducing patient survival rates. The metastatic pathways of HCC are diverse and complex, distinct from those of other malignancies, encompassing direct invasion, extrahepatic dissemination, and intrahepatic spread. Studies have shown that the lungs (up to 60%) and bones (up to 40%) are the most common sites of hematogenous metastasis [3], which may also occur metachronously during treatment or surveillance following curative interventions. Driven by molecular and genetic dysregulation, aggressive tumor cells activate specific signaling pathways—such as the epithelial–mesenchymal transition program—to breach the basement membrane and infiltrate adjacent blood or lymphatic vessels. Once in circulation, these cells disseminate via the bloodstream or lymphatic system to distant tissues, where they ultimately undergo clonal expansion and establish metastatic lesions [4].

The multistep process of metastasis involves intricate molecular events and regulatory mechanisms. Researchers have long been committed to uncovering the molecular mechanisms driving HCC metastasis. These efforts have led to the emergence of targeted therapies, offering novel clinical options, particularly for patients with advanced HCC. In 2007, the tyrosine kinase inhibitor sorafenib was approved, marking a milestone in HCC treatment [5], followed by the clinical application of several other targeted agents [6]. However, the progression of targeted therapies has been accompanied by the growing challenge of drug resistance. This underscores the urgent need to identify potential biomarkers associated with HCC metastasis, which could facilitate early detection and optimize therapeutic strategies.

As our understanding of HCC metastasis deepens, an increasing number of potential biomarkers have been identified, including circulating tumor cells (CTCs), circulating tumor DNA (ctDNA), exosomal contents, and non-coding RNAs (ncRNAs). These biomarkers not only illuminate the biological underpinnings of HCC metastasis but also pave the way for the development of innovative diagnostic tools and targeted therapies.

This review provides a comprehensive overview of the key molecular events and regulatory mechanisms involved in HCC metastasis, including mutations in oncogenes and tumor suppressor genes, the accumulation of epigenetic alterations, and the aberrant activation of critical signaling pathways such as Wnt/β-catenin, TGF-β/Smad, EGFR/PI3K/Akt/mTOR, JAK/STAT, Hippo, Notch, and Hedgehog, all of which play pivotal roles in driving metastatic progression. Advances in the understanding of three-dimensional (3D) genomic architecture abnormalities in metastasis are also summarized. In addition, the review highlights the +currently targeted therapies for advanced HCC and potential biomarkers associated with metastasis. Ultimately, this work aims to offer new perspectives for targeted intervention and biomarker discovery in HCC metastasis, thereby advancing the development of more precise, individualized therapeutic strategies to improve patient outcomes.

## 2. Key Molecular Events and Regulatory Mechanisms in Hepatocellular Carcinoma Metastasis

### 2.1. Genetic Alterations

Cancer gene mutations and expression changes are prevalent in the metastasis of various cancers [7]. Genetic variations influence tumorigenesis and progression, Playing a critical role in enhancing the metastatic potential of tumor cells. These modifications encompass proto-oncogenes, tumor suppressor genes, and essential regulators of cellular processes, including proliferation, apoptosis, and differentiation. While metastatic sites and primary tumors often share common mutations in key driver genes or transcriptional programs, they typically acquire new characteristics during the processes of dissemination and treatment. Previous studies have indicated that, during HCC metastasis, most of the driver genes are clonal mutations, present in every metastatic cell [8]. The spatiotemporal multi-omics evolutionary map of liver cancer metastasis has identified three tumor suppressor genes—*TP53* (32/72), *RB1* (11/72), and *PTEN* (7/72)—as significantly recurrent mutations in extrahepatic metastatic tumors (MT). In addition, both widespread and localized somatic copy number alterations in MT have been observed, including copy number gains at 1q, 8q, and 5p15.33 (*TERT*), as well as copy number losses at 16q, 4p, 17p13.1 (*TP53*), and 9p21.3 (*CDKN2A*). These alterations exhibit a strong correlation with the findings in primary tumors [9]. Therefore, based on the frequent mutations of genes that arise from clonal mutations during the development of the primary tumor, and integrating studies of these mutated genes in metastasis, common mutated genes frequently observed in HCC metastasis include *OPN* [10], *CTNNB1* [11], *ARID1A* [12], *ARID2* [13], *CCND*1 [14], *AXIN1* [15], among others. These alterations facilitate the progression and metastasis of HCC by modifying cellular functions and the tumor microenvironment, thereby exerting a profound influence on the biological behavior of HCC.

*TERT* functions as the catalytic subunit and rate-limiting enzyme of telomerase, responsible for regulating telomere length and influencing cellular proliferation [16]. Research has demonstrated that hotspot mutations in the *TERT* promoter (*TERT*p) (C228T and C250T) are significantly increased during malignant transformation [17]. Furthermore, *TERT*p and *TP53* co-mutation are significantly enriched in patients with positive microvascular invasion, closely correlating with poor prognosis by promoting HCC cell proliferation and inhibiting immune cell infiltration in the surrounding tumor microenvironment [18]. *OPN* is a pivotal gene in promoting HCC metastasis. Research indicates that intracellular or nuclear *OPN* regulates epithelial–mesenchymal plasticity, thereby increasing the population of CSCs and enhancing the tumor’s invasive and metastatic potential [19]. *CTNNB1* encodes β-catenin, a critical intracellular signaling factor in the Wnt pathway, playing an essential role in tumorigenesis by regulating cell growth, adhesion, and differentiation [11]. Studies have shown that activating mutations in *CTNNB1* are closely associated with atypical pro-metastatic mechanisms, including alpha-fetoprotein (AFP) loss [20]. The *CCND1* gene encodes cyclin D1, a proto-oncogene that plays a crucial role in the progression and metastasis of multiple cancers, including HCC [21].

Tumor suppressor genes are crucial in maintaining chromosomal stability, promoting cell differentiation and senescence, and regulating cell proliferation to inhibit tumor growth, acting as key factors in preserving genomic integrity. *TP53*, as a tumor suppressor gene, facilitates DNA repair, induces cell cycle arrest, and accelerates cellular senescence and apoptosis. It also prevents tumorigenesis by regulating tumor metabolism [22]. However, mutations in *TP53* can lead to centrosome amplification, aneuploid cell proliferation, and chromosomal instability [23]. Moreover, these mutations are closely associated with alterations in the tumor immune microenvironment, potentially promoting cancer cell metastasis and invasion [24]. *ARID1A* is a critical tumor suppressor that exerts its function by interacting with transcription factors and their co-repressor complexes, particularly recruiting chromatin remodeling activity [25]. Reduced expression of *ARID1A* is significantly associated with tumor progression, metastasis, and poor overall survival in both human and murine models [12]. *ARID2*, a critical component of chromatin remodeling complexes, is integral to various biological processes and is often found to be mutated in HCC. Studies show that *ARID2* expression is markedly reduced in HCC metastatic tumors and exerts inhibitory effects during the metastasis process [13]. *AXIN1*, another tumor suppressor gene, is associated with the development of various cancers. Recent studies reveal that overexpression of *AXIN1* negatively regulates HCC cell proliferation, migration, invasion, and metastasis, significantly suppressing tumor progression [15]. The known gene changes, their role in HCC progression, and the regulatory pathways are listed in Table 1.

### 2.2. Epigenetic Abnormalities

Epigenetics refers to the alteration in gene activity without changes to the underlying DNA sequence, resulting in phenotypic variations that can be passed down to subsequent generations. In cancer, epigenetic abnormalities encompass multiple aspects of chromatin biology. Numerous studies have extensively examined the pivotal roles of various epigenetic modifications, such as DNA methylation, histone modifications, and microRNAs (miRNAs), in the proliferation and metastasis of HCC [47]. These modifications regulate the epigenetic control of oncogenes and tumor suppressor genes, thereby influencing and impacting the processes of HCC proliferation and metastasis.

#### 2.2.1. DNA Methylation

DNA methylation is a crucial epigenetic modification mechanism, involving alterations in the quantity and pattern of methylation, without changes to the underlying DNA sequence [48]. Abnormal methylation, encompassing both hypomethylation and hypermethylation, is observed in HCC and other common cancers. Specific promoter hypermethylation and global hypomethylation are respectively linked to the inactivation of tumor suppressor genes and genomic instability. Region-specific hypermethylation facilitates tumor progression by silencing tumor suppressor genes [49]. Abnormal expression of DNA methyltransferases (DNMTs), particularly their upregulation, leads to alterations in DNA methylation. These changes are often associated with poor prognosis in patients [50]. Extensive research has shown that DNMT-mediated epigenetic modifications play a central role in regulating the development, metastasis, and invasion of HCC [51]. Notably, several studies have highlighted the involvement of DNMT1 in facilitating HCC metastasis. In specific subpopulations of HCC cells (e.g., CD133+/CD44+), non-collagenous bone matrix protein osteopontin (*OPN*) accumulates, and knockdown of *OPN* significantly inhibits spheroid formation and the expression of stemness-related genes. Further research has revealed that *OPN* enhances the metastatic potential of HCC by regulating DNA methylation [52]. Hepatocyte growth factor (HGF), a multifunctional cytokine, plays a crucial role in the growth and differentiation of normal liver cells through its interaction with the receptor c-Met. However, their upregulation is closely associated with tumor progression and metastasis in HCC. Studies have shown that in the HCC microenvironment, the epigenetic upregulation of c-Met is tightly linked to tumor progression and metastasis. An in-depth analysis of the c-Met promoter reveals that, during hematogenous metastasis in HCC, the increased expression of c-Met in CTCs is directly associated with a significant decrease in DNA methylation levels [53]. Additionally, another significant study further elucidated the role of HGF in the metastatic progression of HCC. HGF induces DNMT1, altering its expression and subsequently leading to the hypermethylation of certain tumor suppressor genes, such as myosin heavy chain-related coiled-coil protein, pannexin 2, and LIM homeobox 9 [54]. Meanwhile, during the invasion and metastasis of HCC, the expression of DNMT3 is also altered, thereby influencing the progression of the disease. A recent clinical–pathological investigation revealed a significant association between the expression profile of DNMTs and survival outcomes in HCC patients. Specifically, within the DNMT family, members demonstrated differential expression patterns with critical prognostic implications. Notably, DNMT3b exhibited marked upregulation in HCC tissues, with its mRNA levels exceeding those in non-neoplastic liver tissues by more than fourfold. This transcriptional hyperactivation correlated with significantly shortened overall survival (OS) and metastasis-free survival (MFS) periods in affected individuals [48]. Multivariate analysis further confirmed that elevated DNMT3b expression served as an independent predictor of unfavorable prognosis, emphasizing its potential role in tumor progression and metastatic dissemination.

#### 2.2.2. Histone Modifications

In recent years, the dysregulation of histone epigenetic modifiers has increasingly been associated with HCC. In the intricate process of tumor progression, histone modifications, particularly methylation, acetylation, and ubiquitination, play a pivotal role [55]. These modifications finely regulate transcriptional processes, influencing the expression of oncogenes and tumor suppressor genes. In the ongoing development of anticancer therapies, considerable attention has been focused on histone-modifying enzymes, with histone methyltransferases and demethylases emerging as particularly promising molecular targets for therapeutic intervention [56]. Numerous studies on HCC have demonstrated a strong correlation between histone methylation and the regulation of genes involved in metastasis and proliferation. Specifically, to trigger primary metastasis in HCC, several methyltransferases and demethylases target histone H3 at lysines (K) 4 and K36, resulting in conformational alterations that disrupt the balance and distribution between euchromatin and heterochromatin. This modification subsequently upregulates genes involved in the mesenchymal–epithelial transition (MET) [57]. SETDB1, also known as KMT1E, is a methyltransferase primarily responsible for methylating histone H3 at lysine 9 (H3K9), thereby suppressing the expression of related genes [58]. Research has shown that SETDB1 knockout leads to a downregulation of T-lymphoma invasion and metastasis gene 1, which subsequently weakens the epithelial–mesenchymal transition (EMT) process and reduces cell migration and invasion. In contrast, in HCC tissues, SETDB1 expression is positively correlated with Tiam1 levels [59]. Furthermore, the euchromatic histone lysine methyltransferase 2 (G9a, also known as EHMT2) and the heterochromatin protein 1 (SUV39H1) are key enzymes responsible for catalyzing the methylation of histone H3K9, thereby inducing heterochromatin formation. Both enzymes are overexpressed in various cancers [60]. In HCC, upregulation of G9a is closely linked to more aggressive clinical and pathological features, while G9a knockout suppresses HCC cell migration and proliferation [61]. Additionally, reducing the expression of the post-transcriptional regulator microRNA-125b, which targets SUV39H1, can induce cellular senescence and decrease cell migration and metastatic potential [62].

KDM5C [63] and JARID1B [64] are histone demethylases in the JmjC domain-containing protein family, primarily responsible for H3K4 demethylation to suppress gene expression through heterochromatin formation, and they are overexpressed in various cancers. In HCC, the histone demethylases KDM5C and JARID1B are highly expressed in aggressive cancer cells, with their expression levels closely associated with distant metastasis. Specifically, silencing KDM5C and JARID1B leads to the epigenetic silencing of bone morphogenetic protein 7 [65] and phosphatase and tensin homolog (PTEN) [66], thereby reducing the migration, invasion, and wound-healing abilities of cancer cells. In summary, histone modifications play a pivotal role in the progression of HCC. By modulating the activity of these key enzymes, we can effectively influence the biological behavior of cancer cells. These findings offer potential targets for the development of novel therapeutic strategies for HCC.

#### 2.2.3. MicroRNA

miRNAs belong to a highly conserved family of small ncRNAs, typically ranging from 18 to 24 nucleotides in length [67]. These RNA molecules play a critical role in regulating gene expression, with their mechanisms of action being both diverse and complex. On one hand, miRNAs can bind to the promoter regions of target genes, influencing gene expression at the transcriptional level [67]. On the other hand, at the post-transcriptional stage, miRNAs suppress translation by either degrading the target mRNA or binding to its 3′-untranslated region (3′UTR) [68]. Notably, a single miRNA molecule can regulate hundreds of target mRNAs containing the same short recognition sequence, highlighting miRNAs’ extensive influence within gene regulatory networks. Additionally, most mRNAs feature multiple binding sites within their 3′UTRs for various miRNAs, further enhancing the complexity and precision of gene expression regulation [69]. Moreover, studies have confirmed that certain miRNAs play a pivotal role in the onset and progression of human cancers, including the invasion and metastasis of cancer cells [70]. In the progression and metastasis of HCC, miRNAs serve as crucial regulators, profoundly impacting biological behaviors such as proliferation, apoptosis, invasiveness, EMT, angiogenesis, drug resistance, and autophagy [71].

Moreover, numerous critical epigenetic regulators can be post-transcriptionally modulated through miRNAs. For instance, miR-152 exhibits a close interaction with miR-148a and DNMT1. Specifically, when the expression levels of these two microRNAs (miR-152 and miR-148a) deviate from the normal range and show a downregulation, it triggers an increase in DNMT1 expression, revealing an inverse relationship between their expression levels [72]. Furthermore, the dysregulation of miRNAs is closely linked to tumor progression and metastasis. Transforming growth factor (TGF)-β signaling plays a pivotal role in the EMT process. Specifically, the activation of the TGF-β pathway induces epithelial cells to lose their inherent characteristics and transition into cells exhibiting mesenchymal traits, a process known as EMT [73]. This transformation is typically associated with increased tumor invasiveness and metastatic potential, as mesenchymal-like cells exhibit enhanced migratory and invasive capabilities. Aberrant miRNA expression, particularly miRNAs that regulate TGF-β signaling or EMT-related genes, may further impact tumor progression and metastasis. Recent scientific studies have revealed that the dysregulation of miRNAs in the TGF-β/Smad signaling pathway can drive the development of malignant tumors. In HCC cases, a consistent downregulation of miR-542-3p and miR-142 expression has been observed. These two miRNAs specifically bind to the 3′-UTRs of their target genes, directly regulating TGFB1 mRNA levels, thereby exerting an inhibitory effect on the TGF-β/Smad signaling pathway [74,75]. This regulatory mechanism is closely linked to the EMT process and the metastatic behavior of HCC. Furthermore, it has been reported that miRNAs also regulate Smad7, a negative regulator of TGF-β signaling. Specifically, the overexpression of miR-216a/217 directly targets Smad7 and the tumor suppressor gene PTEN, thereby promoting EMT, enhancing cell migration, and inducing stem cell-like characteristics [76], all of which contribute to the recurrence and progression of HCC. Research by Wang et al. demonstrated that miR-122, when overexpressed, precisely targets Wnt1 and inhibits the expression of genes associated with EMT, significantly reducing cell proliferation, migration, and invasion [77]. Furthermore, miR-148a has been shown to target the 3′-UTR of Wnt1, exerting a negative regulatory effect on Wnt1 expression in HCC. By inhibiting Wnt1 expression, both miR-122 and miR-148a effectively attenuate the Wnt signaling pathway [78], thereby suppressing HCC tumor progression driven by EMT and cancer stem cell-like properties.

### 2.3. Signaling Pathway Dysregulation

During the metastatic progression of HCC, not only are numerous genetic mutations observed, but multiple signaling pathways also undergo dysregulation. HCC metastasis is driven by the aberrant activation of several key signaling pathways that regulate critical cellular processes, such as proliferation, survival, migration, and invasion. These pathways are not only involved in the initiation of tumorigenesis but also play a significant role in the progression and metastasis of HCC.

#### 2.3.1. Wnt/β-Catenin Pathway

**Figure 1:**
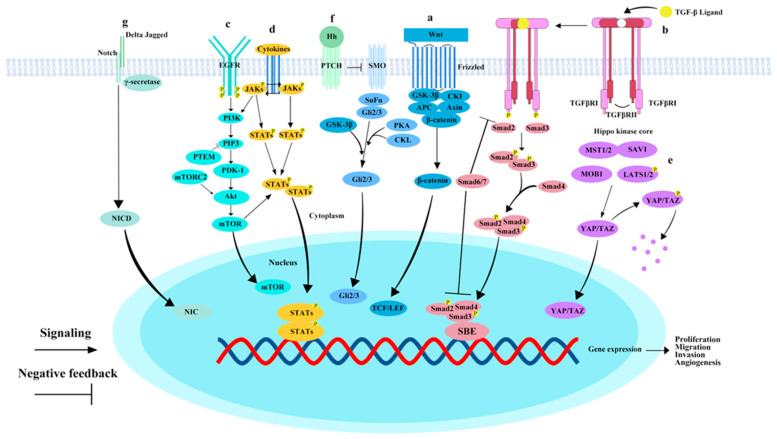
Key pathways involved in hepatocellular carcinoma metastasis. (**a**) Wnt/β-catenin pathway: Upon activation of the canonical Wnt/β-catenin pathway, Wnt proteins bind to receptors, forming a complex that recruits degradation complexes to the cell membrane. This results in the accumulation of β-catenin in the cytoplasm and its subsequent translocation to the nucleus. (**b**) TGF-β/Smad Pathway: The binding of the TGF-β ligand to TGFβRI leads to the phosphorylation of TGFβRI, after which SMAD2 and SMAD3 form a transcriptional complex with SMAD4. This complex then translocates to the nucleus, where it binds to DNA and regulates the expression of target genes in collaboration with various cofactors. SMAD6 and SMAD7 act as negative regulators in the TGF-β/Smad signaling pathway. (**c**) EGFR/PI3K/AKT/mTOR pathway: EGFR binds to ligands such as EGF, causing receptor dimerization and phosphorylation. Phosphorylated EGFR recruits PIK3, which, once activated, phosphorylates membrane lipid PIP2. PIP2 subsequently binds and activates Akt. PTEN dephosphorylates PI3K lipid products, negatively regulating the PI3K/AKT/mTOR pathway. (**d**) JAK/STAT pathway: Various cytokines and growth factors activate the JAK/STAT pathway. Activated JAKs phosphorylate each other and subsequently phosphorylate STAT3 molecules. Phosphorylated STAT3 forms dimers that translocate to the nucleus, acting as transcription factors to regulate gene expression. (**e**) Hippo-YAP/TAZ pathway: MST1/2 kinases bind SAV1 to form a complex that phosphorylates LATs1/2. Phosphorylated LATs1/2 inhibit the transcriptional coactivators YAP/TAZ, confining them to the cytoplasm or promoting their degradation. When the Hippo-YAP/TAZ pathway is inhibited, YAP/TAZ translocates to the nucleus, where they interact with transcription factors such as TEAD to initiate gene expression. (**f**) Hedgehog (Hh) pathway: Hh ligands bind to the transmembrane receptor Ptch, releasing the inhibition on SMO. Activated SMO initiates a signaling cascade in the cytoplasm, ultimately activating the Gli transcription factors. (**g**) Notch pathway: Jagged or Delta-like ligands bind to the Notch receptor, inducing structural changes. γ-secretase mediates receptor cleavage, releasing the Notch intracellular domain (NICD). NICD translocates to the nucleus and binds transcription factor complexes, activating downstream gene transcription. Raf family, initiating a signal transduction cascade. “P” stands for phosphorylation.

The Wnt/β-catenin pathway, comprising Wnt proteins, Wnt ligands, and regulatory proteins, orchestrates critical cellular functions such as proliferation, differentiation, migration, and apoptosis, thereby maintaining cellular homeostasis. Activation of the canonical Wnt/β-catenin pathway involves the binding of Wnt proteins to their receptors, triggering the accumulation of β-catenin in the cytoplasm. This β-catenin then translocates into the nucleus (Figure 1a). The nuclear accumulation of β-catenin is closely associated with elevated levels of epithelial cell adhesion molecules (EpCAM) in subpopulations of HCC, endowing tumor cells with self-renewal capacity, invasiveness, distant metastatic potential, and the ability to initiate early tumor recurrence [79]. Dysregulation of Wnt/β-catenin signaling represents a pivotal genetic alteration in HCC, with studies revealing its strong association with stemness, progression, metastasis, and drug resistance in HCC. Stemness is a primary contributor to tumor recurrence, metastasis, and chemotherapy resistance [80], and the Wnt/β-catenin pathway is intricately linked to CSC characteristics, serving as a crucial signaling pathway for CSC proliferation. Research has demonstrated that Cripto-1 enhances the stemness of HCC cells by stabilizing dishevelled-3, thereby activating the Wnt/β-catenin pathway and promoting the stem-like properties of HCC [81]. Furthermore, β-catenin overexpression significantly boosts the self-renewal ability and tumorigenic potential of HCC CSCs [82]. HCC, various ncRNAs have been identified as regulators of the Wnt/β-catenin signaling pathway, exerting either positive or negative effects, thereby influencing HCC invasion and metastasis. For instance, LncRNA-SNHG7 upregulates the Wnt/β-catenin pathway by binding to miRNA-425, which enhances the proliferation, migration, and invasion potential of HCC cells [83]. LncRNA SOX9 antisense RNA 1 exerts multifaceted effects, not just controlling SOX9 expression but also encouraging EMT by altering the Wnt/β-catenin pathway, which speeds up the tumor cells’ malignant development [84].

#### 2.3.2. TGF-β/Smad Pathway

The TGF-β pathway serves a multifaceted and dual function in the initiation, progression, and metastasis of HCC. Initially, TGF-β signaling primarily mediates its tumor-suppressive actions by inducing processes such as cell cycle arrest, cellular senescence, autophagy, and apoptosis [85]. TGF-β signaling begins with its binding to the TGFβRII receptor, which activates TGFβRII, leading to its complex formation with TGFβRI, and subsequent phosphorylation of TGFβRI. Phosphorylated TGFβRI facilitates the binding of Smad2 and Smad3 with Smad4, thereby forming a transcriptional complex. The complex is subsequently transported into the nucleus, where it interacts with a range of cofactors to bind to DNA and modulate the expression of specific target genes (Figure 1b) [86]. However, as the malignancy of the tumor intensifies, the function of TGF-β shifts, promoting tumor progression and endowing cancer with more aggressive characteristics, such as EMT, tumor microenvironment remodeling, and immune system evasion by cancer cells [87]. Numerous studies have highlighted the significant impact of the TGF-β pathway on HCC metastasis. Research by Luo et al. found that TGF-β1 and RELN exhibit an antagonistic relationship in regulating HCC cell migration, where the suppression of reelin expression by TGF-β1 promotes HCC cell migration. Reelin, a large secreted ECM glycoprotein, has its expression in HCC inhibited, which correlates with high recurrence rates [88]. Moreover, TGF-β drives HCC progression by elevating the levels of lysyl oxidase-like 2, thereby facilitating the remodeling of the cytoskeleton, the formation of vascular-like structures, and enhancing local invasiveness [89].

#### 2.3.3. EGFR/PI3K/AKT/mTOR Pathway

The EGFR/PI3K/AKT/mTOR pathway plays a critical role in regulating various cellular processes, including proliferation, survival, migration, and metabolism. Additionally, it is intricately involved in shaping the tumor microenvironment. Moreover, this pathway is closely linked to therapeutic responses and tumor metastasis [90]. Upon binding with ligands such as EGF, EGFR undergoes dimerization and phosphorylation. The phosphorylated EGFR then recruits PI3K, which, upon activation, converts the membrane lipid phosphatidylinositol 4,5-bisphosphate (PIP2) into PIP3. PIP3, phosphorylated by PI3K, binds to the serine/threonine kinase AKT. Activated AKT, by phosphorylating and inhibiting the activity of the tuberous sclerosis complex (TSC), leads to TSC suppression and subsequent activation of mTOR (Figure 1c). Research indicates that EGFR overexpression occurs in more than half of HCC patients, correlating significantly with tumor metastasis, poor survival rates, and high invasiveness [91]. In the advanced stages of HCC, the activity of the EGFR/PI3K/AKT/mTOR pathway is strongly associated with vascular invasion, poor differentiation, and reduced survival rates [92]. PTEN, a key tumor suppressor, inhibits AKT activation by converting PIP3 into PIP2. Microarray analysis has shown that the extent of PTEN loss and the upregulation of p-mTOR and p-Akt are significantly correlated with vascular invasion, intrahepatic metastasis, and tumor grading [93]. Studies have demonstrated that overexpression of VCP promotes HCC cell proliferation, migration, and invasion by activating the PI3K/AKT/mTOR pathway [94]; furthermore, in a mouse orthotopic cancer model, treatment with INK128, an mTOR inhibitor, displayed remarkable efficacy, effectively inhibiting the progression of HCC and reversing its metastatic trend [95].

#### 2.3.4. JAK/STAT Pathway

The JAK/STAT signaling pathway regulates various cellular processes, including proliferation, stem cell maintenance, differentiation, and immune and inflammatory responses [96], Additionally, it can activate EMT and contribute to the establishment of an immunosuppressive microenvironment, playing a crucial role in the generation and maintenance of CSCs [97]. STAT transcription factors are central mediators in the JAK/STAT signaling cascade. In HCC, STAT3 is a key oncogenic driver, with its phosphorylated form detected in approximately 60% of HCC cases, closely correlating with tumor aggressiveness [98]. Various cytokines act as ligands, triggering the JAK/STAT pathway upon their activation. These factors can initiate the JAK/STAT signaling cascade, promoting HCC invasion and metastasis (Figure 1d). For example, IL-27 predominantly mediates the regulation of inflammation within the tumor microenvironment through the downstream JAK/STAT3 pathway, thereby influencing tumor cell invasiveness [99]. In HCC cells, IL-10 is released to activate the STAT3 pathway in NK cells, suppressing their cytotoxic activity and ultimately leading to HCC recurrence and metastasis [100]. Furthermore, in HCC, the expression of miR-200b-3p is markedly elevated and is transmitted to M0 macrophages via exosomal mechanisms, inducing their transformation into the M2 phenotype. This M2 polarization activates the JAK/STAT signaling pathway, thereby enhancing the proliferation and migration capacity of HCC cells. In this process, the JAK/STAT pathway serves as the critical downstream mediator in the exosome-driven regulation of macrophage polarization by miR-200b-3p and the promotion of HCC metastasis [101].

#### 2.3.5. Hippo Pathway

A pivotal mechanism in the pathogenesis of HCC is the dysregulation of the Hippo signaling pathway. Specifically, this disruption is considered a critical factor in the onset of liver cancer, particularly HCC. In the Hippo-YAP/TAZ signaling pathway, the MST1/2 kinases assemble a complex with SAV1, which then activates LATs1/2. The LATs1/2 kinases phosphorylate and inhibit the transcriptional co-activators YAP/TAZ. When the Hippo-YAP/TAZ pathway is inhibited, YAP/TAZ translocates to the nucleus, where it interacts with transcription factors like TEAD1-4, thereby activating the expression of target genes (Figure 1e). The key components of the Hippo pathway—such as LATS1/2, MST1/2, YAP, and TAZ—can influence tumor metastasis via two main mechanisms. First, the Hippo pathway regulates EMT, impacting the cellular behavior of tumor cells and promoting their migration and invasion [102]. Studies have shown a close link between PDLIM1 and the regulation of HCC metastasis through the Hippo pathway. Mutations at specific amino acid sites in PDLIM1, such as Asn145, disrupt its regulatory role in Hippo signaling and HCC metastasis. PDLIM1 modulates Hippo signaling to inhibit HCC metastasis [103]. Furthermore, certain microRNAs enhance HCC metastasis and EMT by directly inhibiting Hippo-YAP/TAZ signaling. For example, miR-1254 targets PAX5, reducing Hippo signaling both in vitro and in vivo. Thereby enhancing HCC cell proliferation, migration, and invasion [104]. Secondly, the Hippo pathway inhibits anchorage-dependent cell death and apoptosis, which are triggered by the loss of cell attachment to the extracellular matrix, thereby facilitating tumor metastasis. Thus, the key components of the Hippo-YAP/TAZ signaling pathway serve as critical regulators in HCC metastasis and hold significant promise as potential therapeutic targets for anti-metastatic strategies.

#### 2.3.6. Hedgehog Pathway

The Hedgehog (Hh) signaling pathway consists of Hh ligands, transmembrane receptor proteins (Ptch and Smo), Gli transcription factors, and their associated downstream target genes, serving as a pivotal regulatory axis driving the progression of HCC. Upon binding of the Hh ligand to the transmembrane receptor Ptch, the release of Smo is triggered, allowing Smo to enter the cytoplasm and activate the Gli transcription factors, which in turn regulate the expression of specific downstream genes (Figure 1f). Tumor metastasis is a multi-step process in which the Hh pathway may participate directly or indirectly by influencing EMT and MET, or by altering the pre-metastatic niche [105]. Chen et al. conducted a study demonstrating that USP44 suppresses the expression of programmed death ligand 1 (PDL1) in HCC through the downregulation of the Hh signaling pathway. Moreover, the Gli1 inhibitor GANT61 exhibits synergistic effects with anti-PDL1 therapy, further highlighting the potential therapeutic value of the Hh pathway in HCC treatment [106]. The relationship between the Hh signaling pathway and CSCs has become a significant area of research. Zhang et al. demonstrated that the expression of HMGCR positively correlates with the Smoothened receptor in the Hh signaling pathway, promoting the nuclear translocation of the transcriptional activator GLI family zinc. This process represents a key step in the activation of the Hh pathway, influencing the malignant behaviors of CSCs, including proliferation, migration, and invasion [107]. Furthermore, He et al. found that circZNF609 may activate the Hh pathway by repressing the expression of miR-15a-5p and miR-15b-5p, reducing their repressive effect on GLI2, thereby enhancing the stemness of HCC cells and significantly increasing their ability to metastasize to distant sites [108].

#### 2.3.7. Notch Pathway

This signaling mechanism is dependent on intercellular communication. When transmembrane ligands on adjacent cells bind to their corresponding transmembrane receptors, the TNF-α-converting enzyme (also known as the metalloproteinase ADAM, or TACE) mediates the cleavage of the functional extracellular domain (NECD) from the transmembrane intracellular domain (TM-NICD). Within the receiving cell, γ-secretase further cleaves TM-NICD, releasing the NICD fragment. The released NICD translocates to the nucleus, where it interacts with a transcription factor complex to activate classical Notch target genes, including P21, Myc, and members of the HES family, thereby initiating downstream signaling cascades (Figure 1g). It has been established that the Notch pathway can promote EMT in cancer cells. Recent studies have emphasized the critical role of the Notch pathway in HCC metastasis. For example, SORT1 promotes HCC angiogenesis and systemic metastasis by activating the Notch pathway and upregulating CD133 expression [109]. DTL activates the Notch pathway, accelerating HCC cell proliferation, invasion, and resistance to sorafenib [110]. Furthermore, UBE2C contributes to the activation of Notch signaling, thereby enhancing the metastatic potential of HCC cells [111]. Given the growing body of research confirming the significant impact of the Notch pathway in HCC progression and metastasis, targeted therapeutic approaches aimed at this pathway have shown considerable promise.

#### 2.3.8. The Signaling Pathway Network and Interactions Associated with the Metastasis of HCC

In summary, the onset and metastasis of HCC are regulated by multiple signaling pathways, each playing a pivotal role in tumor proliferation, migration, invasion, and metastasis. The Wnt/β-catenin pathway, by regulating the characteristics of cancer stem cells, facilitates HCC metastasis and drug resistance, with its aberrant activation closely linked to the invasiveness of HCC. The TGF-β/Smad pathway exhibits a dual role in HCC, possessing tumor-suppressive functions while simultaneously promoting tumor progression and metastasis through the induction of EMT and remodeling of the tumor microenvironment. The EGFR/PI3K/AKT/mTOR pathway is intricately involved in cell proliferation, survival, and tumor metastasis. Activation of the JAK/STAT pathway in HCC promotes tumor cell migration and metastasis, contributing to immune evasion and further exacerbating the malignancy of the tumor. The Hippo pathway influences HCC metastasis potential by regulating EMT and the loss of cell adhesion, while the Hedgehog pathway drives distant metastasis through EMT mediation and tumor microenvironment modulation. Finally, the Notch pathway promotes HCC invasiveness and metastasis by regulating tumor cell proliferation, migration, and EMT. The aberrant activation of these pathways provides a crucial molecular foundation for the metastasis of HCC.

The metastasis of HCC is driven by the intricate interplay and crosstalk of multiple signaling pathways. These pathways not only function independently but also collaborate through mutual regulation to promote tumor cell proliferation, migration, invasion, immune evasion, and angiogenesis. The Wnt/β-catenin, Raf/MAPK, and PI3K/AKT signaling pathways constitute a complex network that plays a pivotal role in the onset and progression of HCC [112]. Furthermore, the EMT process is a critical determinant in the invasion and metastasis of various cancers, induced by TGF-β through β-catenin signaling [113]. The PI3K/AKT/mTOR pathway, by regulating cell proliferation and survival, collaborates with the TGF-β/Smad pathway to augment tumor invasiveness [114]. The JAK/STAT, Hippo, Hedgehog, and Notch pathways also play vital roles in regulating cancer stem cell properties, promoting EMT, and facilitating tumor metastasis. In summary, the synergistic interplay among these signaling pathways drives HCC metastasis and malignant progression, offering potential targets for research and therapeutic intervention.

### 2.4. Alterations in Three-Dimensional Genomic Architecture

The study of the aberrant 3D genome structure in cancer has emerged as a promising area of research in recent years. Recent research evidence indicates that the 3D genome architecture is closely linked to DNA replication, transcriptional activity, repair mechanisms, gene expression and regulation, embryonic development, and the onset of diseases [115]. Furthermore, alterations in 3D genome architecture have been observed in the metastatic progression of HCC. Utilizing high-throughput chromosome conformation capture technology (Hi-C), scientists have gained a deeper understanding of chromatin’s 3D morphology and its internal interactions, enabling the precise identification and delineation of 3D genomic structures. These structures are classified based on the size characteristics of chromosomal fragments into chromatin territories (CT), compartment divisions, topologically associating domains (TADs), and chromatin loops (CL) [116].

Recent studies have revealed that higher-order chromosomal structural alterations, such as chromosomal interactions, compartment reprogramming, and the fragmentation or fusion of TADs, are implicated in cancer development [117]. Studies have shown that compared to normal liver tissue, there are significant changes in the interactions within and between chromosomes in HCC. Additionally, tumor-specific TAD boundaries and enhancer hijacking have been identified in HCC tissues [115]. The disruption of TAD boundaries exerts a profound impact on gene expression and disease development, contributing not only to cancer initiation but also playing a crucial role in tumor metastasis. Research indicates that the hepatitis B virus (HBV) can influence the formation of specific TAD boundaries, altering those with lower insulation [118], which subsequently affects the expression of genes associated with cell adhesion, thereby promoting HCC metastasis. Moreover, some studies have reported that when *ARID1A* is defective, BRG1 expression is reduced, impairing the function of the BRG1-RAD21 axis. This dysfunction prevents RAD21 from effectively maintaining the structural stability of TADs, leading to TAD remodeling and disruption of chromatin loops. In liver cells with ARID1A deficiency, the number of TADs decreases. This TAD conformational change induced by *ARID1A* deficiency further affects the expression of genes related to HCC cell invasion, enhancing their invasive capability [119]. Moving forward, the application of 3D genomics technologies, such as Hi-C, ATAC-seq, and ChIA-PET, to explore the dynamic changes in TAD abnormalities during the development and metastasis of HCC may provide novel strategies for targeted therapy in HCC.

## 3. Targeted Therapies Associated with HCC Metastasis

The metastasis of HCC marks the progression of the tumor to an advanced stage, typically signifying a more complex disease course and a substantial increase in treatment difficulty. Traditional therapeutic approaches, including surgical resection, radiation therapy, chemotherapy, and interventional treatments, yield limited efficacy for HCC patients with recurrence or metastasis. However, with the growing understanding of the molecular mechanisms underlying HCC, targeted therapies have progressively become a vital treatment strategy for patients with advanced HCC. Sorafenib remains the standard first-line treatment for HCC, while newer targeted therapies such as lenvatinib, bevacizumab, and donafenib have been approved, providing a broader range of first-line treatment options. A landmark clinical trial in 2017 confirmed that regorafenib significantly extended overall survival in patients whose disease progressed following sorafenib resistance, heralding the introduction of second-line therapies. Subsequently, the approval of ramucirumab and cabozantinib further enriched the therapeutic landscape, offering advanced HCC patients a more diverse array of treatment options.

### 3.1. First-Line Targeted Therapy

#### 3.1.1. Sorafenib

In the European multicenter SHARP trial, patients with unresectable HCC and Child–Turcotte–Pugh grade A cirrhosis were randomly assigned to either the sorafenib or placebo group. The results showed that the median overall survival (mOS) for patients in the sorafenib group was 10.7 months, significantly higher than the 7.9 months observed in the placebo group (HR 0.69, 95% CI 0.55–0.87). Moreover, the imaging progression-free survival (PFS) in the sorafenib group was also significantly extended, from 2.8 months to 5.5 months [120]. In a multicenter, double-blind, randomized Phase III trial conducted in the Asia–Pacific region, the study included HCC patients with Child–Pugh grade A liver function who had not previously received systemic therapy. These patients were randomly assigned to either the sorafenib or placebo group. The results indicated that, compared to the placebo group, sorafenib treatment significantly prolonged the median overall survival (6.5 months vs. 4.2 months; hazard ratio 0.68, 95% CI 0.50–0.93). Additionally, the time to radiological progression was significantly extended, from 1.4 months to 2.8 months [121].

The GIDEON trial demonstrated the favorable therapeutic efficacy of sorafenib in patients with unresectable hepatocellular carcinoma. The results indicated that among the intention-to-treat population (n = 3213), the mOS of Child–Pugh A patients was significantly higher than that of Child–Pugh B patients (13.6 months vs. 5.2 months), while the median time to progression (mTTP) was comparable (4.7 months vs. 4.4 months) [122]. The U.S. FDA has designated sorafenib as the first-line treatment for inoperable or advanced HCC [123]. These studies further affirmed sorafenib’s significant efficacy and favorable tolerability in treating advanced HCC, particularly in patients with metastatic liver cancer.

#### 3.1.2. Lenvatinib

Lenvatinib is a potent oral multi-targeted tyrosine kinase inhibitor that exerts significant anticancer activity through the suppression of various kinases, including VEGFR1–3, PDGFR, KIT, FGFR1–4, and RET. Furthermore, Lenvatinib reduces the levels of matrix metalloproteinases (MMPs 1, 2, 7, 9, 10, and 16) while enhancing the expression of tissue inhibitors (TIMPs 1, 3, and 4), thereby significantly restraining the invasive and metastatic potential of HCC cells [124]. In 2018, both the U.S. FDA and China’s NMPA approved lenvatinib as a first-line targeted therapy for patients with unresectable hepatocellular carcinoma. In a randomized, phase III non-inferiority trial with a crossover design, the study data revealed that the lenvatinib group demonstrated statistically significant non-inferiority in overall survival, with a median overall survival of 13.6 months, notably surpassing the 12.3 months observed in the sorafenib group. In terms of therapeutic efficacy, lenvatinib exhibited superior disease control, achieving a median progression-free survival of 7.4 months, a median time to progression of 8.9 months, and an objective response rate of 24%, compared to 3.7 months, 3.7 months, and 9%, respectively, in the sorafenib group. Regarding predefined secondary efficacy endpoints, lenvatinib consistently outperformed sorafenib in treatment response rates. Safety analyses indicated no significant differences in drug tolerability between the two groups, with comparable rates of treatment-related adverse events and treatment discontinuation due to adverse effects (13% vs. 9%) [125]. Taken together, the survival benefits and safety profile of lenvatinib substantiate its efficacy as a potent therapeutic option for advanced HCC.

#### 3.1.3. Bevacizumab

Bevacizumab (BVZ) is a humanized recombinant IgG1 monoclonal antibody specifically designed to bind to VEGF, thereby preventing its interaction with receptors and neutralizing VEGF’s biological activity [126]. The results of the International IMbrave150 Phase III randomized clinical trial demonstrated the safety and efficacy of this combination. The findings revealed that the atezolizumab (ATZ) + BVZ group had a higher OS rate compared to the sorafenib group (67.2% vs. 54.6%) and a superior median PFS (68 months vs. 63 months) [127]. The combination of ATZ + BVZ has emerged as the current standard first-line treatment for patients with unresectable or metastatic HCC.

#### 3.1.4. Donafenib

Donafenib is a targeted therapy derived from the structural optimization of sorafenib, capable of inhibiting the activity of multiple targets, including VEGFR, PDGFR, and RAF, thereby preventing tumor growth and angiogenesis [128]. A Phase II/III clinical study demonstrated that the donafenib treatment group showed a significant advantage in OS compared to the sorafenib control group. Patients treated with donafenib achieved an mOS of 12.1 months, surpassing the 10.3 months observed in the sorafenib group, representing a 17% reduction in the risk of disease progression. In terms of safety profile, the incidence of adverse events was largely comparable between the two groups [129]. In June 2021, donafenib was officially approved for market release in China as a first-line treatment for patients with unresectable or metastatic advanced HCC.

### 3.2. Second-Line Targeted Therapies

#### 3.2.1. Regorafenib

Regorafenib is a second-line therapeutic agent for HCC, functioning as a multi-kinase inhibitor that targets various pathways involved in tumor progression. By inhibiting key signaling nodes such as VEGFR, FGFR-1, PDGFR, KIT, RET, and B-RAF, it effectively suppresses tumor growth. In a Phase III trial that was randomized, double-blind, and placebo-controlled [130], 573 individuals with Barcelona stage B or C hepatocellular carcinoma, who had shown disease progression following sorafenib therapy, were included. Survival analysis revealed that the mOS in the regorafenib group was 10.6 months, significantly surpassing the 7.8 months observed in the placebo group, indicating a notable survival benefit. Additionally, other experimental studies have demonstrated that regorafenib alleviates portal hypertension, angiogenesis, and liver cirrhosis [131]. Future research will explore its potential in combination therapies with other drugs. Given the lack of alternative treatments for patients progressing on sorafenib, regorafenib appears to address this unmet need.

#### 3.2.2. Cabozantinib

Cabozantinib, a small-molecular-weight tyrosine kinase antagonist, shows specific inhibition of several receptors essential in tumor growth and metastasis, such as VEGFR1, VEGFR2, VEGFR3, MET, and AXL [132]. In a double-blind Phase III trial, 707 patients with advanced HCC who experienced disease progression following sorafenib treatment were enrolled. The results showed that the mOS in the cabozantinib group was 10.2 months, significantly longer than the 8.0 months observed in the placebo group. Median progression-free survival was 5.2 months in the cabozantinib group, compared to 1.9 months in the placebo group [133]. In early 2019, the U.S. FDA granted official approval of cabozantinib, positioning it as a novel second-line therapeutic choice for patients with advanced HCC [134].

#### 3.2.3. Ramucirumab

Ramucirumab is a monoclonal antibody targeting VEGFR-2, effectively inhibiting the binding of VEGF to its receptor, thereby suppressing tumor angiogenesis and metastasis [135]. A Phase III clinical study demonstrated that ramucirumab significantly improved PFS and OS in certain patients with HCC, particularly those with elevated AFP levels (≥400 ng/mL). In this subgroup, the mOS in the ramucirumab group reached 8.5 months, notably higher than the 7.3 months in the control group (*p* = 0.0199), while the mPFS was extended from 1.6 to 2.8 months (*p* < 0.0001). In terms of safety, ramucirumab was well tolerated, with grade ≥3 adverse events primarily consisting of hypertension (12.2%) and hyponatremia (5.6%) [136]. These findings suggest that ramucirumab provides a survival benefit with an acceptable safety profile in advanced HCC patients with elevated AFP levels who have failed sorafenib treatment.

#### 3.2.4. Apatinib

Apatinib is a tyrosine kinase inhibitor targeting VEGFR-2, primarily exerting its antitumor effects by inhibiting tumor angiogenesis. Studies have shown that apatinib effectively suppresses the VEGF–VEGFR signaling pathway, thereby inhibiting the growth, invasion, and migration of residual tumor cells within the hypoxic microenvironment following embolization [137]. In a Phase III clinical trial involving 400 patients with advanced HCC, survival analysis revealed that the mOS in the apatinib group reached 8.7 months, significantly longer than 6.8 months in the placebo group. The mPFS was also extended from 1.9 to 4.5 months. These findings confirm that apatinib effectively reduces the risk of disease progression in patients with advanced HCC who have failed prior chemotherapy or targeted therapies, offering a new therapeutic option for this difficult-to-treat population [138]. Table 2 provides a summary of first- and second-line targeted therapies for advanced HCC since 2008.

## 4. Potential Biomarkers Associated with HCC Metastasis

Previously, we explored the key molecular events and regulatory mechanisms underlying HCC metastasis. The metastatic progression of HCC is closely linked to multiple factors, and research into these factors has led to the identification of several molecules strongly associated with HCC metastasis. These molecules show great potential as biomarkers, offering valuable insights for the prediction, diagnosis, and prognosis of HCC metastasis.

### 4.1. Potential Metastasis-Related Biomarkers Based on Genetic Mutations

Genetic mutations play a crucial role in the metastatic progression of HCC, involving alterations in several key genes. The exploration of genetic mutation-based biomarkers has become a significant focus in the research and clinical management of metastatic HCC. He et al. divided HCC patients into two groups: single tumor and multiple tumors or metastasis. They analyzed the mutation allele frequencies (MAF) of genes in plasma cfDNA across both groups. The results revealed that patients with multiple tumors or HCC accompanied by metastasis exhibited significantly higher mutation frequencies in TP53, RET, APC, and FGFR3 compared to those with single tumors, suggesting that these mutations are closely associated with HCC metastasis [139]. These mutations hold potential as predictive biomarkers for early diagnosis of metastasis. Sun et al. focused on clonal mutations to identify recurrently mutated genes in metastatic tumor tissues. Their study identified three tumor suppressor genes—TP53, RB1, and PTEN—that were significantly recurrent in metastatic tissues [9], indicating their potential as novel predictive biomarkers for metastasis. Furthermore, the study found that mutations in the TP53 gene within exosomal DNA (exoDNA) were significantly associated with microvascular invasion (MVI) in HCC patients. In a cohort of 60 patients, 80% (48 cases) showed the TP53 c.747 G > T mutation in exoDNA. Multivariate logistic regression analysis revealed a strong correlation between TP53 mutations and the occurrence of MVI (*p* < 0.01). Further, receiver operating characteristic (ROC) curve analysis identified an optimal TP53 mutation frequency cutoff of 67% for predicting MVI [140]. These findings suggest that TP53 mutation frequency in exoDNA can serve as an effective biomarker for predicting microvascular invasion in HCC. Overall, genetic mutation-based biomarkers offer an essential direction for the early diagnosis and personalized treatment of HCC metastasis.

### 4.2. Potential Metastasis-Related Biomarkers Based on Epigenetic Abnormalities

Epigenetic modifications profoundly influence the expression patterns of metastasis-associated genes at the molecular level. These alterations not only provide valuable insights into the mechanisms of cancer metastasis but also offer new possibilities for developing dynamic biomarkers for monitoring blood or tissue samples. In HCC, epigenetic factors such as miRNAs, DNA methyltransferases/demethylases, and histone methyltransferases/demethylases have been shown to be closely associated with HCC metastasis. Biomarkers based on these epigenetic abnormalities hold significant potential as tools for predicting and diagnosing HCC metastasis.

#### 4.2.1. Methylation-Based Potential Biomarkers for HCC Metastasis

The metastasis of HCC is closely associated with gene methylation. Studies have shown that hypermethylation of the promoter region of the DEUP1 gene is strongly correlated with lymph node metastasis and tumor differentiation [141]. Similarly, hypermethylation of the ASS1 gene promoter is tightly linked to the invasiveness and migratory potential of HCC [142], indicating its promise as a potential diagnostic and therapeutic target in the context of HCC metastasis. The promoter hypermethylation of the APC gene is especially evident in the later stages of HCC and is associated with lymph node metastasis and tumor size [143], suggesting its potential as a prognostic biomarker for HCC metastasis. Moreover, hypermethylation of the MT1M and MT1G gene promoters is closely linked to vascular invasion and metastasis, highlighting their potential as prognostic indicators for HCC metastasis [144]. In conclusion, gene methylation holds significant promise for the early detection and personalized treatment of HCC metastasis, offering remarkable potential as a metastasis-associated biomarker.

#### 4.2.2. Histone Modification-Based Potential Biomarkers for HCC Metastasis

Histone modifications play a critical role in the metastasis of HCC. Overexpression of histone H3 (H3K27me3) is closely associated with tumor vascular invasion, tumor size, metastasis, and poor prognosis. Furthermore, ROC curve analysis evaluating the prognostic value of H3K27me3 expression in predicting the survival status of HCC patients demonstrated an encouraging predictive value, with an area under the curve (AUC) of 0.733. In an independent validation cohort, H3K27me3 continued to show promise as a predictor of survival status in HCC patients, with an AUC of 0.719 [145]. These findings indicate that H3K27me3 is not only significant among metastasis-related biomarkers but also serves as a potent prognostic marker for HCC. The histone demethylases JARID1B and KDM5C, members of the JMJC family, are capable of demethylating H3K4 and are overexpressed in HCC and other types of cancer. In HCC, the high expression of KDM5C and JARID1B significantly increases in infiltrating cells and correlates closely with distant metastasis. Silencing or knockout of KDM5C and JARID1B has been shown to inhibit the epigenetic network regulating PTEN and BMP7 [146], significantly suppressing HCC migration and invasion. These findings reveal a close connection between the abnormal processes of histone acetylation and deacetylation and HCC tumor proliferation and poor clinical outcomes. Furthermore, they suggest that the analysis of epigenetic markers may offer a feasible diagnostic method for the early detection and prognostic evaluation of HCC metastasis.

#### 4.2.3. Epigenetic Modification-Based miRNA Potential Biomarkers for HCC Metastasis

Numerous studies have demonstrated that miRNAs play a pivotal regulatory role in the metastasis of HCC, holding significant potential as biomarkers for metastasis diagnosis and prediction. Various miRNAs are closely associated with HCC invasion, metastasis, TNM staging, prognosis, and postoperative recurrence [147]. Among the members of the let-7 family, the upregulation of let-7a, let-7c, and let-7e is strongly linked to serosal invasion, venous invasion, and advanced TNM stages. Notably, high expression of let-7c is associated with significantly shortened overall survival after surgery, indicating that let-7 miRNAs not only participate in HCC progression but also correlate with poor prognosis, making them potential biomarkers for metastasis prediction [148]. MiR-148a-3p demonstrates strong efficacy in distinguishing metastatic from non-metastatic HCC patients (AUC = 0.800, sensitivity 88.89%, specificity 60.0%), with its downregulation correlating with elevated TGF-β1, distant metastasis, multinodular disease, and advanced TNM stages, highlighting its potential as a diagnostic biomarker for HCC metastasis [149]. MiR-652-3p promotes EMT and HCC metastasis by inhibiting TNRC6A expression, presenting itself as a potential prognostic biomarker for metastatic HCC. Additionally, ref. [150] high expression of miR-130b in HCC is closely associated with tumor number, vascular invasion, and TNM stage, making it a crucial predictor of postoperative recurrence and metastasis. Serum miRNAs also show promise in non-invasive diagnostics [151]. Increased expression of miRNA-96-5p correlates with tumor size and metastatic spread, whereas reduced levels of miRNA-99a-5p are strongly associated with metastasis. Combined analysis with AFP in ROC curve testing shows a high AUC of 0.97, surpassing the individual markers, indicating the potential for their combined use in HCC metastasis diagnosis. Furthermore, ref. [152] the preoperative serum expression levels of miR-497 and miR-1246 are significantly correlated with tumor differentiation, TNM staging, and metastasis. The combination of AFP with miR-497 and miR-1246 demonstrated the strongest diagnostic capability for distinguishing HCC, with an AUC of 0.955 (95% CI: 0.837–0.958), sensitivity of 94.0%, and specificity of 86.0%, further suggesting their potential as diagnostic biomarkers for metastasis.

In terms of HCC metastasis prognosis, miRNAs also hold substantial promise as prognostic biomarkers [153]. MiR-501-3p is significantly downregulated in metastatic HCC cell lines and recurrent tumor tissues, with its reduced expression closely associated with tumor progression and poor survival outcomes. Similarly, ref. [154] miR-187-3p shows low expression in HCC tissues and cell lines, correlating with advanced TNM stage and metastasis, especially under hypoxic conditions, where it is significantly downregulated and promotes metastasis and EMT, underscoring its potential as a prognostic marker [155]. Low expression of miRNA-1179 is linked to higher rates of lymph node and distant metastasis, predicting poorer survival prognosis.

In conclusion, miRNAs, as epigenetic modifiers, play a crucial role in the onset and progression of HCC metastasis. These associated miRNAs not only hold great potential as non-invasive diagnostic and metastasis predictive biomarkers but also provide important insights for personalized treatment and prognosis evaluation. Table 3 summarizes the key functions and associated features of the aforementioned miRNAs in HCC metastasis.

### 4.3. Potential Metastasis-Related Biomarkers in HCC Based on Aberrant Signaling Pathways

Abnormalities in signaling pathways play a pivotal role in the initiation, progression, and metastatic invasion of cancer. Consequently, identifying metastasis-related biomarkers based on signaling pathway abnormalities has become a critical focus in HCC metastasis research.

Among these signaling pathways, Wnt/β-catenin signaling is considered an early signaling event in the pathogenesis of HCC [156]. The Wnt/β-catenin pathway has been identified as a critical signaling cascade driving the proliferation of CSCs. Overexpression of β-catenin not only enhances the self-renewal capacity of HCC CSCs but also increases their tumorigenic potential in vivo [157]. Therefore, β-catenin serves as a pivotal biomarker in the Wnt/β-catenin pathway involved in metastasis, with significant diagnostic and prognostic potential.

The TGF-β/SMAD signaling pathway plays a multifaceted regulatory role in HCC. Studies have demonstrated that MMP-8 and TGF-β1 accumulate in highly invasive HCC cell lines and patient tissues, correlating with changes in the EMT phenotype and the migration and invasion of HCC cells [158]. SMAD4, a key regulator in the TGF-β/SMAD signaling cascade, exerts a complex influence on HCC development [159]. Yuan et al. found that high expression of SMAD4 and TGF-β is significantly associated with lower survival rates in HCC patients [160]. Thus, TGF-β1 and SMAD4 play crucial roles in HCC metastasis through the TGF-β/SMAD signaling pathway, establishing them as important biomarkers for monitoring HCC metastasis.

The EGFR/PI3K/AKT/mTOR signaling cascade is strongly associated with HCC metastasis [90]. EGFR overexpression is observed in 68% of HCC cases and correlates with metastasis, poor patient survival, and aggressive tumor characteristics [91]. Furthermore, EGFR ligands are also overexpressed in human liver cancer cells and tumor tissues, particularly in advanced HCC [161]. Consequently, EGFR, as a pivotal molecule in this pathway, holds significant potential as a predictor of HCC metastasis. In certain HCC subtypes, the aberrant activation of the JAK/STAT pathway leads to the dysregulation of downstream target genes, impacting various cellular functions [162]. STAT3 enhances the invasive potential of HCC cells by modulating the expression of genes such as MMP-2 and MMP-9 [163]. Furthermore, STAT3 promotes tumor invasion by upregulating proteins involved in EMT [164]. These findings highlight STAT3 as a key regulatory factor in HCC progression, influencing multiple aspects of tumorigenesis and metastasis, and underscore its potential as a biomarker for metastasis.

The transcriptional co-activators YAP and TAZ, essential downstream effectors of the Hippo signaling pathway, are central to its dysregulation. The activation of YAP and TAZ is strongly associated with the progression and metastasis of HCC [164]. YAP activation is associated with aggressive malignant behaviors such as cell proliferation, stemness, and EMT [165,166]. Research by Hyunjin Park et al. demonstrated that the expression of YAP and TAZ in HCC correlates with invasive clinical and pathological features, stemness, and EMT [167]. Therefore, YAP/TAZ serves as a significant biomarker for the aggressive biological behavior of HCC, holding substantial clinical value. Hh signaling participates in cancer metastasis through both direct and indirect mechanisms, potentially acting via EMT, MET, or pre-metastatic niche formation [168]. Hedgehog signaling components, including SHh, PTCH1, and GLI1/2, are frequently upregulated in tumor tissues or bodily fluids; they serve as an indicator of cancer progression [169]. A detection method based on aptamer sensors indicates that SHh exhibits high specificity in distinguishing HCC from adjacent non-tumorous tissues, particularly in AFP-negative patients, where increased SHh levels compensate for the limitations of AFP detection. Increased SHh expression is associated with a higher incidence of portal vein invasion, indicating its promise as a predictive marker for early metastatic events in HCC. Finally, Notch signaling is critically involved in the initiation, development, invasiveness, and neovascularization of various cancers [170]. Within this pathway, the receptor Notch1 is markedly overexpressed in HCC and is linked to increased tumor dimensions, metastatic presence, and microvascular infiltration [171]. The significance of Notch1 in HCC metastasis underscores its potential as a metastasis-related biomarker.

In conclusion, the aberrant activation or suppression of signaling pathways plays a crucial role in the metastasis of HCC. These critical signaling pathways and their related molecular components enhance our comprehension of HCC metastasis while providing important guidance for early detection, prognostic assessment, and individualized therapeutic approaches.

### 4.4. Potential Emerging Biomarkers Implicated in HCC Metastasis

#### 4.4.1. Circulating Tumor Cells as Potential Biomarkers of HCC Metastasis

Driven by advances in minimally invasive techniques, liquid biopsy has recently gained prominence in the field of cancer biomarker research, enabling non-invasive analysis of biomarkers through body fluids. By examining CTCs, tumor-derived products such as DNA, and exosomal contents in the bloodstream, it becomes possible to dynamically monitor the genomic landscape of tumors [172].

CTCs originate from primary tumor sites or metastatic lesions and can be detected in peripheral blood circulation. These cells are considered potential indicators of postoperative recurrence and metastasis. Rather than representing a uniform clone, CTCs constitute a heterogeneous population derived from various tumor lesions, capable of altering their phenotypic and molecular characteristics in response to selective microenvironments and therapeutic pressures [173]. Chen et al. demonstrated that CTCs can distinguish between metastatic and non-metastatic HCC patients. In patients with metastatic disease or advanced-stage Barcelona Clinic liver cancer (BCLC), mesenchymal phenotype CTCs were significantly elevated [174], suggesting their potential as diagnostic markers of HCC metastasis. Further investigations by Court et al. identified a vimentin-positive CTC subpopulation, strongly associated with advanced or metastatic HCC [175]. These findings underscore the potential clinical relevance of mesenchymal CTCs—especially those expressing vimentin—as biomarkers in HCC metastasis research. Thaninee Prasoppokakorn employed an immunoaffinity-based approach utilizing EpCAM and mucin-1 to detect CTCs. The study revealed that higher CTC counts in peripheral blood were correlated with more aggressive tumor features and poorer survival outcomes in HCC patients [176], highlighting their promise as novel prognostic biomarkers. Currently, CTC detection faces challenges such as limited sensitivity and specificity, extremely low abundance in blood, difficulty in early detection, and high tumor heterogeneity. These limitations hinder its widespread application in early cancer diagnosis [177]. Nevertheless, since CTCs originate from primary or metastatic sites and carry molecular information reflecting tumor heterogeneity, mutations, and drug resistance, they hold significant value in metastasis-related biomarker research. Amplification of cancer-associated genes in CTCs via reverse transcription PCR can aid in identifying metastatic signatures.

#### 4.4.2. Circulating Tumor DNA as a Potential Biomarker for HCC Metastasis

In cancer patients, uncontrolled cell death in tissues and organs releases a substantial amount of nucleic acids into the circulation, forming ctDNA. As ctDNA originates from tumor cells, its analysis provides insights into the heterogeneous collection of tumor cells carrying mutations and epigenetic patterns [178]. Research by Cai et al. revealed that the HCK (p.V174M) mutation site is associated with HCC recurrence and metastasis, promoting cell migration and invasion [179]. This mutation is found at elevated levels in ctDNA and holds promise as a potential biomarker for forecasting HCC metastasis and prognosis [180]. Li et al. discovered that elevated methylation of ctDNA linked to the Wnt/β-catenin signaling pathway is strongly associated with tumor size, TNM staging, distant metastasis, and HBV infection in liver cancer. This suggests that the methylation level of Wnt/β-catenin pathway-related ctDNA could serve as a promising biomarker for HCC metastasis with substantial diagnostic potential. Furthermore, studies indicate that the presence of ctDNA is closely associated with the prediction of recurrence and extrahepatic metastasis [181]. Among 46 patients, the cumulative incidence of recurrence and extrahepatic metastasis was significantly higher in the ctDNA-positive group compared to the ctDNA-negative group (*p* = 0.0102 and *p* = 0.0386, respectively). Additionally, multivariate analysis identified ctDNA as an independent predictor of portal vein microvascular invasion (OR 6.10, 95% CI, 1.11–33.33, *p* = 0.038), further supporting its potential in predicting HCC metastasis.

Similar to CTCs, the clinical application of ctDNA also faces challenges. Identifying ctDNA within the vast pool of free DNA from other cell types presents significant technical hurdles. Moreover, it remains uncertain whether ctDNA can accurately reflect the genetic composition of rapidly growing and highly mobile tumor cells.

#### 4.4.3. Exosomal Contents as Potential Biomarkers for HCC Metastasis

Exosomes, extracellular vesicular aggregates loaded with different biomolecules, play a major role in the initiation, advancement, and spread of HCC [182]. A growing body of evidence suggests that the contents of exosomes hold significant potential as novel biomarkers associated with HCC metastasis. These components mainly include proteins, miRNAs, lncRNAs, and circular RNAs (circRNAs) [183]. Exosomal proteins, which are highly abundant in HCC-derived exosomes, play a pivotal role in the mechanisms driving HCC invasion and metastasis. Sun et al. revealed that exosomal protein S100A4 significantly enhances HCC metastatic potential by inducing the upregulation of OPN and multiple metastasis-related genes, as well as activating the STAT3 phosphorylation pathway [184]. Therefore, S100A4 is regarded as a novel prognostic biomarker and a potential therapeutic target associated with HCC metastasis. Moreover, the SMAD3 protein contained in exosomes released by HCC can be transported to distal sites or surrounding tissues. This transfer facilitates the survival and proliferation of CTCs after they settle at distant locations, thereby accelerating the metastatic process of HCC. Consequently, the detection of SMAD3 protein in serum exosomes typically signals the potential occurrence of metastasis [185].

In addition to exosomal proteins, non-coding RNAs identified in plasma-derived exosomes represent a novel class of potential biomarkers associated with HCC metastasis.Clinical evidence reveals that increased plasma levels of exosomal miR-92a-3p are markedly correlated with shorter OS and PFS in HCC patients, highlighting its potential as an indicator of unfavorable prognosis. ROC analysis demonstrated an AUC of 0.8534 (*p* < 0.001) for miR-92a-3p in distinguishing metastatic from non-metastatic HCC patients, further validating its potential as a predictive biomarker for HCC metastasis [186]. Research by Hye Seon Kim et al. found that exosomal miR-125b is markedly reduced in patients with metastatic HCC and shows a strong association with extrahepatic spread and clinical outcomes [187]. These findings suggest that exosomal miR-125b serves as a robust predictor of early extrahepatic metastasis in HCC. Another study showed that serum exosomal miR-34a is notably decreased in HCC patients and is strongly associated with clinical characteristics. Reduced miR-34a levels were significantly associated with tumor differentiation, TNM staging, tumor invasion depth, and lymph node metastasis (*p* < 0.05). ROC analysis indicated that the combined detection of serum exosomal miR-34a and AFP has high diagnostic capability in distinguishing HCC patients from healthy controls [188], indicating its promise as a non-invasive biomarker for early detection of HCC and monitoring of metastasis. One such exosomal lncRNA, TUG1, is a target of miR-524-5p and is secreted by cancer-associated fibroblasts (CAFs). In human patients, the elevated expression of TUG1 in metastatic HCC plays a pivotal role in HCC metastasis [189], indicating its potential to predict the metastasis of HCC. Additionally, research has demonstrated that the expression level of serum exosomal circRNA-100338 could serve as a biomarker for predicting lung metastasis following radical liver resection in HCC patients [190]. Furthermore, exosomal circRNA-SORE enhances the migration and invasion potential of low-metastatic HCC cell lines via the circPTGR1-miR-449a-MET regulatory axis, indicating its potential as a biomarker for monitoring HCC progression [191]. These studies highlight the potential of small RNA molecules derived from serum exosomes as valuable biomarkers for the prediction and diagnosis of HCC metastasis in clinical settings.

**Figure 2:**
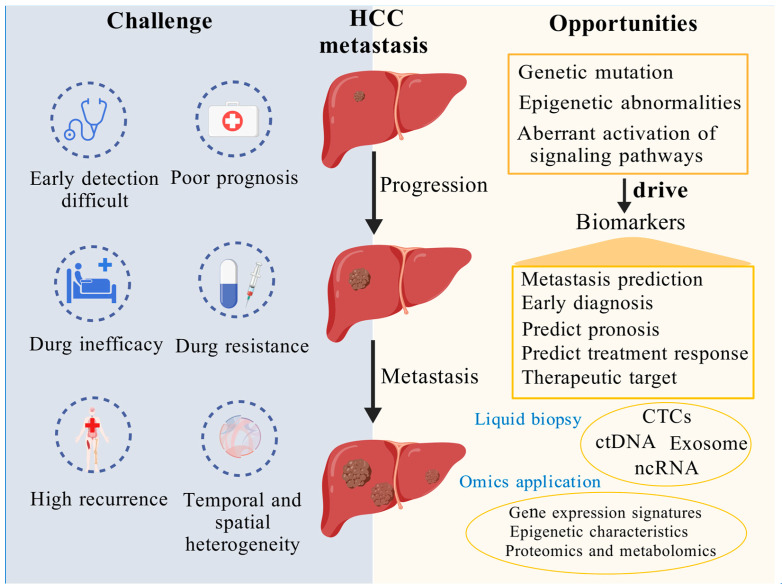
Exploring and investigating potential biomarkers of HCC metastasis offers a promising pathway to enhance early detection and enable more precise, targeted therapeutic strategies. This figure underscores the critical importance of identifying and exploring potential biomarkers associated with HCC metastasis. On the left, it illustrates the major challenges posed by HCC metastasis, including difficulties in early detection, poor prognosis, drug inefficacy, resistance, high recurrence rates, and spatiotemporal heterogeneity. On the right, it highlights promising opportunities to address these challenges through the discovery of metastasis-related biomarkers driven by genetic mutations, epigenetic alterations, and aberrant activation of signaling pathways. Key biomarkers—such as CTCs, ctDNA, exosomes, and ncRNA—can be detected through liquid biopsy and analyzed using multi-omics technologies to enable metastasis prediction, facilitate early diagnosis, refine prognostic assessments, evaluate therapeutic responses, and identify targeted treatment strategies. Created using BioGDP.com (accessed on 3 April 2025) [193].

HCC metastasis is an extraordinarily intricate biological process, involving continuous protein and metabolic interactions between tumor cells and their surrounding microenvironment. While traditional serum markers, such as AFP, are widely employed for HCC screening, their limitations in monitoring the specificity and dynamic changes in tumor metastasis are evident [192]. In recent years, significant advancements in proteomics and metabolomics have made protein and metabolic biomarkers essential tools for elucidating the mechanisms of HCC metastasis and guiding precision therapies. These biomarkers provide real-time insights into tumor cell metabolic reprogramming, extracellular matrix remodeling, and immune evasion states. Figure 2 illustrates the critical importance of identifying and exploring potential biomarkers associated with HCC metastasis.

## 5. Conclusions

This review provides a comprehensive analysis of the mechanisms underlying HCC metastasis, delving into the roles of key genetic mutations, epigenetic alterations, and the aberrant activation of signaling pathways in HCC progression and metastasis, with particular emphasis on the impact of 3D genome structural changes on gene expression regulation. Additionally, the review summarizes current first- and second-line targeted therapies for advanced HCC and explores potential biomarkers associated with HCC metastasis, such as CTCs, ctDNA, and exosomal contents. These biomarkers hold significant promise for early warning, diagnosis, and prognostic prediction in HCC metastasis.

As research into the mechanisms of HCC metastasis deepens, an increasing number of critical molecular events have been uncovered. These include driver gene mutations, epigenetic abnormalities, aberrant activation of key signaling pathways, and dynamic remodeling of the 3D genomic structure. These molecular mechanisms not only enhance our understanding of the biological nature of HCC metastasis but also offer new perspectives for early detection, precision treatment, and prognostic evaluation. With the continued advancement of targeted therapies, treatment strategies are becoming increasingly precise, with drugs specifically targeting particular gene mutations, epigenetic alterations, and key signaling pathways. These emerging targeted therapies hold the potential to significantly improve treatment efficacy, delay metastatic progression, and enhance patient survival outcomes.

Moreover, the in-depth exploration of these molecular mechanisms has laid the theoretical foundation for the discovery of related biomarkers, further advancing the application of novel biomarkers such as liquid biopsy, gene expression profiles, and non-coding RNAs. These tools are paving the way for more precise early diagnosis, treatment response prediction, and metastasis monitoring in HCC. Notably, changes in the 3D genomic structure underscore its potential role in transcriptional dysregulation and HCC metastasis, offering a fresh direction for future research into this disease.

A growing body of research emphasizes the critical role of 3D genome structural variations in tumorigenesis, particularly in HCC. Changes in 3D genome structure, such as remodeling of chromatin compartments, topologically TADs, and chromatin loops, play a significant role in the transcriptional imbalance observed in HCC [118]. Compared to normal liver cells, HCC cell lines exhibit notable alterations in chromatin interaction patterns [194], further promoting the invasiveness and metastatic potential of HCC. Additionally, extrinsic factors, such as HBV infection, reshape the chromatin interactions, induce TAD boundary changes, and cause large-scale structural variations, thereby reconfiguring the genomic landscape of hepatocytes and facilitating viral replication and tumor progression [118]. The loss of the tumor suppressor ARID1A has also revealed a direct link between 3D genome structure and HCC metastasis [119]. Although current research has unveiled various patterns of 3D genome remodeling in HCC and their potential oncogenic mechanisms, several challenges remain. These include the assessment of structural consistency across different samples, the lack of functional validation approaches, and the integration of spatial omics data with clinical prognostic information. Future research should continue to explore the role of 3D genomics in cancer, while simultaneously integrating spatial omics with clinical data to advance the precision of personalized treatment and prognostic evaluation.

In future research, a key focus will be on overcoming treatment resistance and enhancing the specificity of targeted therapies, as well as integrating multi-omics data to more accurately predict the early manifestations of metastatic HCC. Furthermore, with the rapid advancement of liquid biopsy technologies, biomarkers such as ctDNA, CTCs, and exosomal RNA will enable more sensitive and specific non-invasive early screening, disease monitoring, and treatment efficacy evaluation. The multidimensional integration of 3D genomics, proteomics, epigenetics, and metabolomics holds great promise for further elucidating the molecular mechanisms of HCC metastasis and providing stronger support for precision medicine.

In conclusion, this review not only summarizes the key mechanisms of HCC metastasis but also anticipates future innovations in targeted therapies and biomarker applications. With the integration of multidisciplinary approaches and technological advancements, we can look forward to the realization of early diagnosis and personalized treatment for HCC metastasis, significantly improving patient survival rates and quality of life.

## Figures and Tables

**Table 1:** Known genetic alterations in HCC metastasis, role in the metastatic process, and regulatory pathways.

Genes	Alteration	Role in HCC Progression	Pathway	References
Proto-oncogenes				
*TERT*	Copy number gain	microvascular invasion (+), immune cell infiltration (−)	NF-κB pathway, Wnt/β-catenin pathway	[18,26]
*CDKN2A*	copy number loss	Immune cell infiltration (−)	MAPK signaling pathway	[9,27]
*OPN*	Overexpression	Metastasis (+)	MAPK, NF-κB, PI3K/Akt	[10]
*CTNNB1*	mutation	Immune escape (+), chemoresistance (+)	WNT/β-catenin	[28]
*CCND1*	Amplification or deletion	Immune escape (+), chemoresistance (+)	TGF-β/Smad	[14]
*FGF1*, *FGF2*, or *FGF19*	Amplification or deletion	Angiogenesis (+), migration (+), invasion (+)	AKT/MTOR, RAS/MAPK, FGF/FGFR	[29]
*Met*	Overexpression	Migration (+), invasion (+)	AKT/MTOR, RAS/MAPK	[30]
*COL4A1*	Overexpression	Migration (+), invasion (+)	FAK-Src	[31]
*SIX4*	Overexpression	Loss of envelope (+), microvascular invasion (+), elevated TNM stage (+), poor prognosis (+)	HGF-SIX4-c-MET	[32]
*CENPU*	Overexpression	Invasion (+), migration (+), cell cycle progression (+)	CENPU/E2F6/E2F1	[33]
*ONECUT2*	Overexpression	Increased tumor number (+), tumor capsule loss (+), microvascular invasion (+), poor tumor differentiation and advanced TNM (+)	FGF2/FGFR1/ONECUT2	[34]
*CENPM*	Overexpression	Immune cell infiltration (+)	P53	[35]
*HOXA9*	Overexpression	Proliferation (+), invasion (+), migration (+), proliferation (−)	RPL38/HOXA9	[36]
*STOML2*	Overexpression	Proliferation (+), invasion (+), migration (+), proliferation (−)	STOML2/PINK1	[37]
*USP11*	Overexpression	EMT (+), metastasis (+)	USP11/eEF1A1/SP1/HGF	[38]
*NET1*	Overexpression	Proliferation (+), invasion (+), migration (+)	Akt	[39]
*GINS1*	Overexpression	Proliferation (+), invasion (+), migration (+), EMT (+)	β-catenin	[40]
Tumor suppressor gene				
*TP53*	Mutations, deletions	Tumor cell stemness (+), vascular invasion (+)	P53 pathway	[24]
*ARID1A*	Mutations or deletions	Lymph node and distant metastases (+)	Epigenetic modifiers Chromatin remodeling	[12]
*ARID2*	Mutations or deletions	Invasion (+), Migration (+)	PI3K/AKT, DNMT1-Snail axis	[13]
*AXIN1*	Mutations or deletions	Proliferation (+), invasion (+), migration (+), EMT (+)	Wnt	[15]
*PTEN*	Mutations or deletions	Invasion (+), metastasis (+)	AKT/mTOR	[32]
*TSC1/2*	mutation	Metastasis (+)	mTOR	[41]
*RB1*	Mutations or deletions	Proliferation (+)	FOXM1-FOXO1 axis	[42]
*APEX1*	Overexpression	Proliferation (+), invasion (+), migration (+)	APEX1/MAP2K6	[43]
*PDE7B*	down-regulated	Proliferation (+), invasion (+), migration (+), EMT (+)	PI3K/AKT	[44]
*FGA*	point mutations	Aggressive (+)	TYK2-STAT3-IL6	[45]
*BAP1*	Overexpression	Migration (+)	CTCF and NRF1/OGT axis	[46]

Abbreviations: +, promote; −, inhibit; TNM, tumor-node-metastasis; EMT, epithelial–mesenchymal transition.

**Table 2:** Clinical application of first- and second-line targeted drugs for the treatment of advanced HCC since 2008.

Targeted Drugs	Targets	Trial Design	NCT Number	Primary Endpoint	Grade 3/4 Drug-Related Side Effects (%, Trial Drug)	Indication	References
First-line treatment							
Sorafenib	VEGFR, PDGFR-β, RAF	Phase III RTC (sorafenib vs. placebo)	NCT00105443	mOS 10.7 vs. 7.9 months	Hand-foot skin reactions (10.7), diarrhea (6.0), fatigue (3.4)	First-line therapy for advanced HCC patients with inoperable or distant	[120]
		Phase III RTC (sorafenib vs. placebo)	NCT00492752	mOS 6.5 vs. 4.2 months	Hand-foot skin reactions (8), diarrhea (8), weight loss (2), hypertension (2)		[121]
Lenvatinib	VEGFR, FGFR, PDGFR, RET, KIT	Phase III RTC (lenvatinib vs. sorafenib)	NCT01761266	mOS 13.6 vs. 12.3 months	Hypertension (42), diarrhea (39), decreased appetite (39), decreased weight (31)	First-line therapy for advanced HCC patients with inoperable or distant metastases	[125]
Bevacizumab	VEGF-A	Phase III RTC (atezolizumab and bevacizumab vs. sorafenib)	NCT03434379	mOS > 17 vs. 13.2 months	Hypertension (15.2), fatigue (2.4), increased AST (7), increased ALT (3.6)	First-line therapy for advanced HCC patients with inoperable or distant metastases	[127]
Donafenib	VEGFR, PDGFR	Phase II/III RTC (donafenib vs. sorafenib)	NCT02645981	mOS 12.1 vs. 10.3 months	Hypertension (9), hand-foot skin reactions (6), diarrhea (2), decreased platelet count (4)	First-line therapy for advanced HCC patients with inoperable or distant metastases	[129]
Second-line treatment							
Regorafenib	VEGFR, FGFR, PDGFR, KIT, RET, B-RAF	Phase III RTC (regorafenib vs. placebo)	NCT0177434	mOS 10.6 vs. 7.8 months	Hypertension (15), hand-foot skin reactions (13), fatigue (9)	Advanced HCC patients who have failed prior first-line therapy	[130]
Cabozantinib	MET, VEGFR, ROS1, RET, AXL, NTRK, KIT	Phase III RTC (cabozantinib vs. placebo)	NCT01908426	mOS 10.2 vs. 8.0 months	Hypertension (16), hand-foot skin reactions (17), fatigue (10), increased AST (12), diarrhea (10)	Advanced HCC patients who have failed prior first-line therapy	[133]
Ramucirumab	VEGFR2	Phase III RTC (ramucirumab vs. placebo)	NCT02435433	mOS 8.5 vs. 7.3 months	Hypertension (13), hyponatremia (6), increased AST (3)	Advanced HCC patients who have failed prior first-line therapy and AFP ≥ 400 ng/mL	[136]
Apatinib	VEGFR2	Phase III RTC (apatinib vs. placebo)	NCT02329860	mOS 8.7 vs. 6.8 months	Hypertension (28), hand-foot skin (18), decreased platelet count (13)	Advanced HCC patients who have failed prior first-line therapy	[138]

Abbreviations: AFP, α-fetoprotein; ALT, alanine aminotransferase; AST, aspartate aminotransferase; mOS, median overall survival. Copyright 2024, the authors of [6].

**Table 3:** Summary of miRNAs as potential biomarkers for HCC metastasis.

miRNA	Primary Function/Role	AUC	Sensitivity (%)	Specificity (%)	Associated Features	References
let-7c	Associated with tumor invasion, metastasis, and TNM staging	--	--	--	High expression is linked to serosal invasion, venous invasion, and advanced TNM stages, particularly let-7c, which is associated with significantly shortened overall survival after surgery.	[147]
miR-148a-3p	Differentiates metastatic from non-metastatic HCC patients	0.800	88.89	60.0	Downregulation is associated with elevated TGF-β1, distant metastasis, multinodular disease, and advanced TNM stages.	[148]
miR-652-3p	Promotes EMT and HCC metastasis by inhibiting TNRC6A	--	--	--	Acts as a potential prognostic biomarker, involved in the metastasis and EMT processes in HCC.	[149]
miR-130b	Closely associated with tumor number, vascular invasion, and TNM staging	--	--	--	High expression is associated with postoperative recurrence and metastasis in HCC.	[150]
miRNA-96-5p	Associated with tumor volume and metastasis	0.82	69.1	85.5	Upregulation enhances diagnostic performance when combined with miRNA-99a-5p and AFP.	[151]
miRNA-99a-5p	Strongly associated with tumor metastasis	0.88	70.9	90.9	Combined with miRNA-96-5p and AFP, it demonstrates the highest diagnostic accuracy for HCC.	[151]
miR-497 + miR-1246 + AFP	Combined for diagnosing and identifying HCC metastasis	0.955	94.0	86.0	Strongest diagnostic capability for distinguishing HCC, with correlations with tumor differentiation, TNM staging, and metastasis after combining with AFP	[152]
miR-501-3p	Downregulated in metastatic HCC cell lines and recurrent tumor tissues	--	--	--	Downregulation is associated with tumor progression and poor survival outcomes.	[153]
miR-187-3p	Low expression in HCC tissues and cell lines, correlating with advanced TNM stage and metastasis	--	--	--	Significantly downregulated under hypoxic conditions, promoting metastasis and EMT, underscoring its potential as a prognostic marker	[154]
miR-1179	Low expression linked to lymph node and distant metastasis	--	--	--	Associated with poor survival prognosis, with low expression predicting lymph node and distant metastasis in HCC	[155]

Abbreviations: miRNA, microRNA; HCC, hepatocellular carcinoma; AUC, area under the ROC curve; --, not reported; TNM, tumor-node-metastasis classification system; TGF-β1, transforming growth factor-beta 1; EMT, epithelial–mesenchymal transition; AFP, alpha-fetoprotein; +, combine.

## Data Availability

Not applicable.
